# A sacrificial layer strategy for photolithography on highly hydrophobic surface and its application for electrowetting devices

**DOI:** 10.1038/s41598-017-04342-z

**Published:** 2017-06-21

**Authors:** Han Zhang, Qiuping Yan, Qingyu Xu, Changshi Xiao, Xuelei Liang

**Affiliations:** 1 Key Laboratory for the Physics and Chemistry of Nanodevices and Department of Electronics, Peking University, Beijing, 100871 China; 2 Department of Physics, Southeast University, Nanjing, 211189 China; 3 School of Navigation, Wuhan University of Technology, Wuhan, 430070 China; 4 Nanjing Jingaowei Optoelectronic Technology Co. Ltd., Nanjing, 210007 China

## Abstract

Patterning micro-structures on highly hydrophobic surface by photolithography is usually inevitable for fabricating devices based on electrowetting effects. The key challenges for such photolithography processes are how to coat photoresist uniformly and maintain the hydrophobicity of the highly hydrophobic surface, which are usually two contradict aspects. Moreover, the patterned microstructure must adhere to the highly hydrophobic surface excellently, which is critical for device application. However, a simple and robust fabrication process that fulfills all the above requirements was seldom reported. In this paper, we developed a sacrificial layer photolithography strategy on highly hydrophobic surface. Photoresist is easily coated uniformly all over the substrate by introducing a sacrificial layer between the photoresist and the highly hydrophobic surface. The hydrophobicity of the exposed hydrophobic surface was maintained and the adhesion of the microstructures to the substrate is excellent. An electrowetting display sample was demonstrated by this fabrication strategy, which showed dynamic image displaying with response time less than 40 ms. The strategy is applicable to both rigid and flexible substrate and manufacturing compatible. We believe our developed photolithography process is important for research and development of devices based on electrowetting effect.

## Introduction

**Figure 1:**
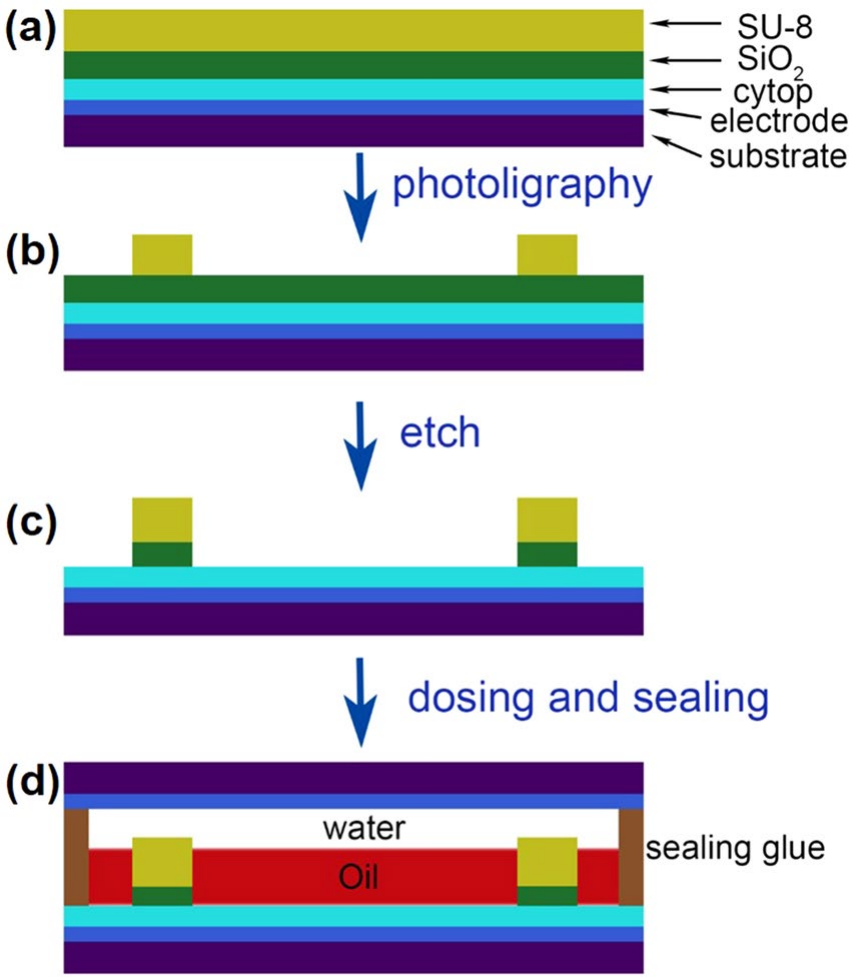
Process flow of the sacrificial layer photolithography strategy on highly hydrophobic substrate (**a**–**c**), and schematic structure of the EWD (**d**).

Electrowetting (EW) is an electromechanical effect which generally refers to changing the contact angle of liquid to a solid surface by application of an electric field^1, 2^. It has become one of the most powerful tools for manipulating tiny amounts of liquids in microsystems. The applications of electrowetting range from ‘lab-on-a-chip’ devices^3–5^ to adjustable liquid lenses^6–8^ and new kinds of electronic displays^9–12^. In these applications, the microsystems are usually fabricated by photolithography on a solid dielectric layer with very low surface energy (highly hydrophobic) and the liquid was confined on this layer by relatively hydrophilic vertical grid or walls^13^. Bottom electrodes were patterned below the hydrophobic dielectric layer, and a top substrate with common or separated electrodes was used to seal the liquid in between. The liquid was manipulated by the electric field that generated between the top and bottom electrodes, as schematic diagramed in Fig. 1d. Usually lower surface energy of the hydrophobic dielectric layer leads to better manipulation of the liquid^2^. However, this also causes more difficulties for fabricating the hydrophilic grid or walls by photolithography to confine the liquid^13–15^.

There are many reports on fabrication of devices based on electrowetting effect. In these reports, highly hydrophobic dielectric materials (fluoropolymer such as Teflon^TM^ AF^16^, CYTOP^13^, Fluoropel^13^, etc.) were usually used for the low surface energy layer. SU-8 negative-tone photoresist is a common choice for the hydrophilic grid. The SU-8 is wet with aqueous liquid and does not dissolve or degrade over time in the presence of water, oil or light^13^. More importantly, SU-8 resist is photo-patternable with easily controlled grid thickness. For the purpose of EW devices, patterning the SU-8 grid on the fluoropolymer must not foul the hydrophobicity of the surface, and the SU-8 grid must adhere to the fluoropolymer with such strength that it survives in a tape test or a finger-nail scratch test^13^. These requirements are very challenging for the EW devices fabrication. Patterning microstructures on fluoropolymer is not an easy task, because photoresists are very difficult to coat on the surface of fluoropolymer due to its high hydrophobicity^13–15^. High viscous photoresists^13, 15, 17, 18^ are possible to be coated on fluoropolymer directly. The hydrophobicity was not compromised in this way, however, coating results of the resist was not satisfying^14, 19^. Photoresist at the edge area of the wafer shrinks towards the center during soft bake of it, which results in serious edge bead and leaving a relatively large blank edge area without resist^14^. Furthermore, the adhesion of the patterned resist at the end of processing was very poor. The fabricated grid may even be blown off the substrate by a nitrogen gun (Supporting information S1). To facilitate the resist coating, various leveling agents (e.g. FluoroN of Cytonix Corp^13^. or Zonyl^®^ FSN^15^) or oxygen plasma^14, 20^ were employed to treat the fluoropolymer, which allows the SU-8 to be spin coated on the fluoropolymer surface with pretty good coverage. However, either the surface hydrophobicity was found to be degraded^14, 15, 20^ or the adhesion was still not good enough^13, 17, 18^.

In spite of pretty good micro-fabrication results of SU-8 grid on fluoropolymer were presented in many reports^6, 12–14^, the complete description of the details of the fabrication process was seldom released, which made the results difficult to repeat by other groups^13^. Therefore, a robust and general fabrication process for electrowetting devices is important for its development and application. In this work, we report a simple and robust fabrication process of SU-8 grid on fluoropolymer surface. SU-8 resist can be coated and patterned uniformly throughout the surface of the fluoropolymer. The patterned SU-8 grid adhered to the substrate every well and the hydrophobicity of the exposed fluoropolymer was almost not influenced. An electrowetting display (EWD) samples were fabricated following our proposed process flow, which demonstrates the potential application of the fabrication process.

## Results and Discussion

**Figure 2:**
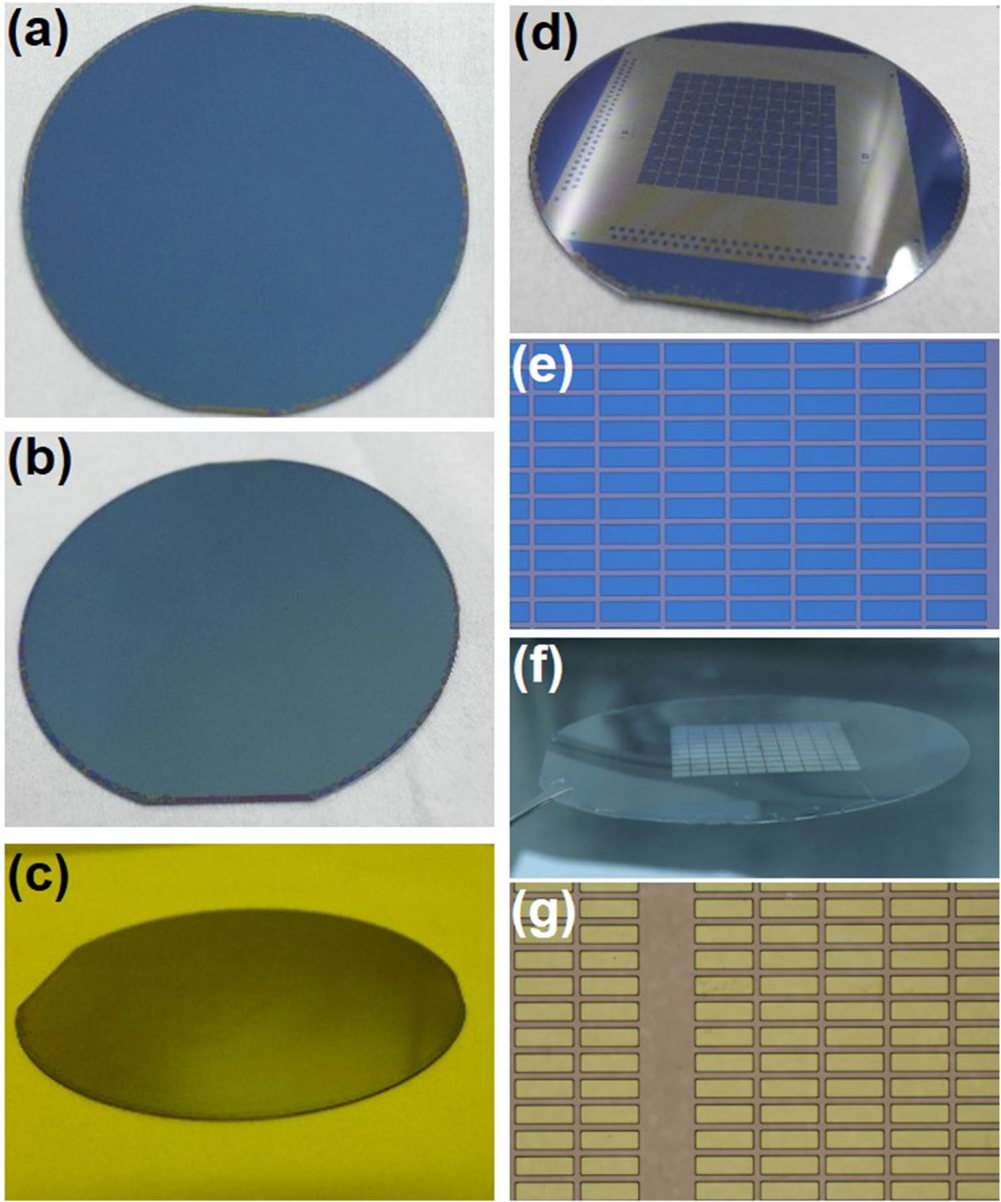
Optical images of the photolithography process. (**a**) CYTOP coated Si wafer, which was hard baked at 180 °C. (**b**) After SiO_2_ deposition. (**c**) After spin coating of SU-8 3010 resist. (**d**) After photolithography and SiO_2_ etching. (**e**) Typical zoom-in image of (**d**). The same process on PET (**f**) and corresponding zoom-in image (**g**). Also see Supporting information S1.

There are several types of commercial available fluoropolymer usually used for electrowetting or micro-fluid devices as mentioned before^13, 15, 16^. In this work, CYTOP (Asahi, CTL-809M) was used as the hydrophobic dielectrics, and the solution was diluted to a concentration about 3%. The CYTOP solution was spin coated onto the silicon wafer at a speed of 1000 rpm for 1 minute. Then it was hard baked on hotplate at 180 °C for 20 minutes to evaporate the solvent. The thickness of the obtained CYTOP film was about 115 nm, which is very uniform except for the very edge area of wafer due to the edge bead effect (Fig. 2a). The water contact angel (CA) of the obtained CYTOP film was measured to be ∼115° in average. Such surface is too hydrophobic for spin coating of photoresist (Supporting information S1).

**Figure3:**
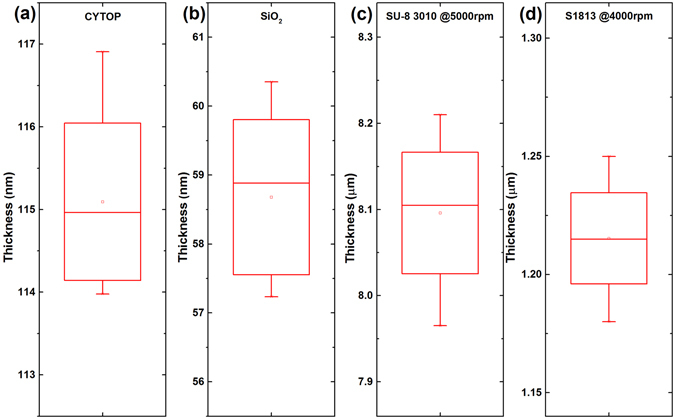
Film thickness measurement around the 4-inch substrate of (**a**) CYTOP, (**b**) SiO_2_ on CYTOP, and the coated high (**c**) and (**d**) low viscous photoresists. The thickness fluctuations of these films are less than ± 3% with 3 mm edge exclusion zone.

As mentioned above, leveling agent or oxygen plasma treatment will facilitate coating of SU-8 on CYTOP, however, with the cost of hydrophobicity^14, 15, 20^, which degrades the performance of the EW devices. To coating SU-8 on CYTOP, we introduced a sacrificial layer strategy as shown in Fig. 1. A layer of SiO_2_ (∼60 nm) was deposited onto hard baked CYTOP by e-beam evaporation. This layer was used as sacrificial layer in the following fabrication steps. The evaporated SiO_2_ covered the CYTOP surface uniformly as shown in Figs 2b and 3b. Then SU-8 3010 (Micro Chem) was spin coated on the SiO_2_ layer, and pre-baked at 95 °C on hot-plate for 3 min. Benefiting from the SiO_2_ sacrificial layer, either high or low viscous photoresist was easily coated on the substrate with 100% coverage (Fig. 2c and Supporting information S1). Then the designed patterns were easily transferred to the substrate by conventional photolithography (Fig. 2d,e). After exposure, the SU-8 resist was post-baked at 95 °C for another 3 min., followed by developing and rinsing. The exposed SiO_2_ was removed by HF etching (1:25 diluted, 30s) (Supporting information Fig. S1.3). Then the sample was rinsed in deionized water and blown dry by nitrogen. Finally, the fabricated SU-8 grid was hard baked at 150 °C for 30 min. to fully crosslink the resist and evaporate the solvent. Following these procedures, SU-8 was easily coated and patterned on both rigid and flexible substrates uniformly (Fig. 2f,g).

The thickness uniformity of the coated photoresist was measured at different locations around the substrate, and the results were shown in Fig. 3. The thickness fluctuation of both high (SU-8 3010) and low (S1813, Microposit) viscous resist are less than ± 3%, which is consistent with the uniformity specification of our spin coater.

**Figure 4:**
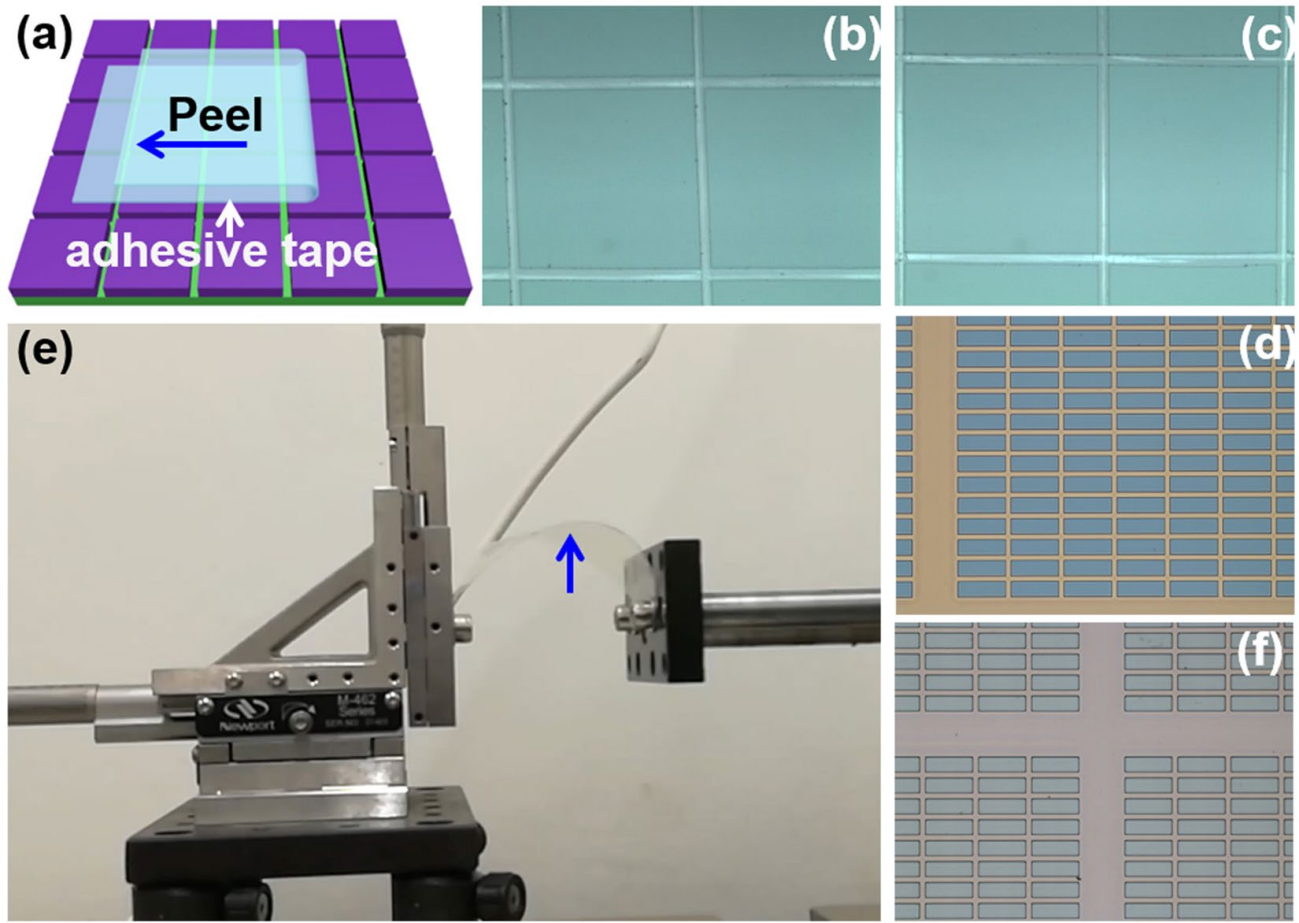
Tape test and bending test for adhesion. (**a**) Schematic illustration of tape test. (**b**) Typical images of tape test of SiO_2_ on CYTOP without and (**c**) with 30 min. annealing at 150 °C. (**d**) Typical tape test results of SU-8/SiO_2_/CYTOP stack on Si wafer. (e) Bending test for structures on flexible substrate. (**f**) Images of the SU-8/SiO_2_/CYTOP stack on PET which was taken at the most strained area (arrow in (**e**)) after bending test. Also see Supporting information S2.

As mentioned before, the adhesion of the fabricated SU-8 grid to the fluoropolymer and the hydrophobicity of the fluoropolymer are critical for the EW devices. To characterize the adhesion, standard tape test^21^ was carried out (Fig. 4a). We firstly tested the adhesion of the evaporated SiO_2_ film to the CYTOP (Supporting information S2). As shown in Fig. 4b, the squares cut in the as-deposited SiO_2_ layer was not peeled off the CYTOP by the adhesive tape. Since there is an annealing step in our process, so the adhesion of SiO_2_ to CYTOP was also tested after 150 °C annealing treatment, and the result was shown in Fig. 4c. These results demonstrate the SiO_2_ sacrificial layer adheres to the CYTOP surface very well. The finally used actual grid structure for EWD devices is the SU-8 on SiO_2_ sacrificial layer as shown in Fig. 1c,d. So tape test was carried out on the fabricated SU-8/SiO_2_/CYTOP stack. Figure 4d shows the optical images of the tested SU-8/SiO_2_/CYTOP stack on Si substrate. The structure was not found affected by peeling of the tape, which demonstrates the adhesion of the SU-8/SiO_2_/CYTOP stack is also very good. The adhesion of the grid structure on flexible substrate (PET) was characterized by a bending test as shown in Fig. 4e (Supporting information S2 and Video-1). After 10,000 times of bending, the fabricated grid structure still kept its integrity and no peeling off the PET substrate was found even in the most strained area (Fig. 4f). These results suggest the adhesion of our fabricated structure can fulfill the requirement of EW devices on both rigid and flexible substrates.

**Figure 5:**
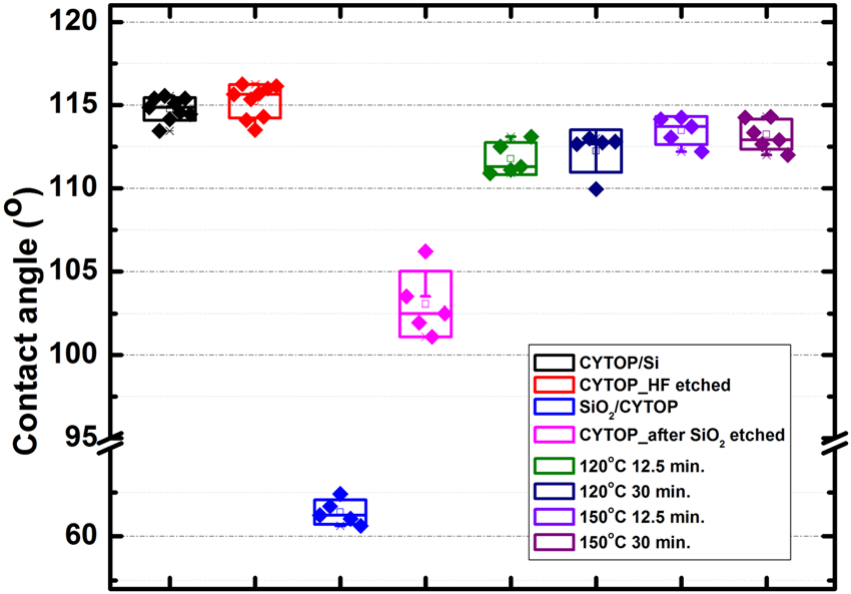
Contact angle measurement results during the photolithography process.

The hydrophobicity change of CYTOP was monitored during the fabrication process, as shown in Fig. 5. The CA of the as-coated CYTOP is ~115°. Since the CYTOP will exposed to HF during the SiO_2_ etching, thus the as-coated CYTOP sample was put into HF solutions for 1 min. to test the influence of the HF etching on the CA of CYTOP. The measurement indicates HF has no effect on the hydrophobicity of the surface of CYTOP. After SiO_2_ deposition, the measured CA of the SiO_2_ surface is ~60°^22^. It is very easy for photoresist, either high or low viscosity, coating on such surface, which gives more choice for the grid materials of the EW devices (Supporting information S1). After the SiO_2_ layer was etched away by HF, the CA of the exposed CYTOP is ~103°. This indicates the hydrophobicity of the CYTOP was slightly affected by the e-beam deposition of SiO_2_. The evaporated SiO_2_ bombarded the CYTOP surface, which changes the surface energy of CYTOP and results in excellent adhesion between SiO_2_ and CYTOP. Though the SiO_2_ evaporation affects the CYTOP slightly, we still want to minimize this effect. Therefore thermal anneal was carried out. As shown in Fig. 5, 120 °C anneal will recover the CA to ~112°, and 150 °C anneal improve the CA to 113°, almost fully recovered. As we specified before, 150 °C hard bake, was used after patterning SU-8 grid. This commonly used hard bake procedure not only hardened the SU-8 grid, but also recovered the hydrophobicity of the exposed CYTOP surface in our process.

**Figure 6:**
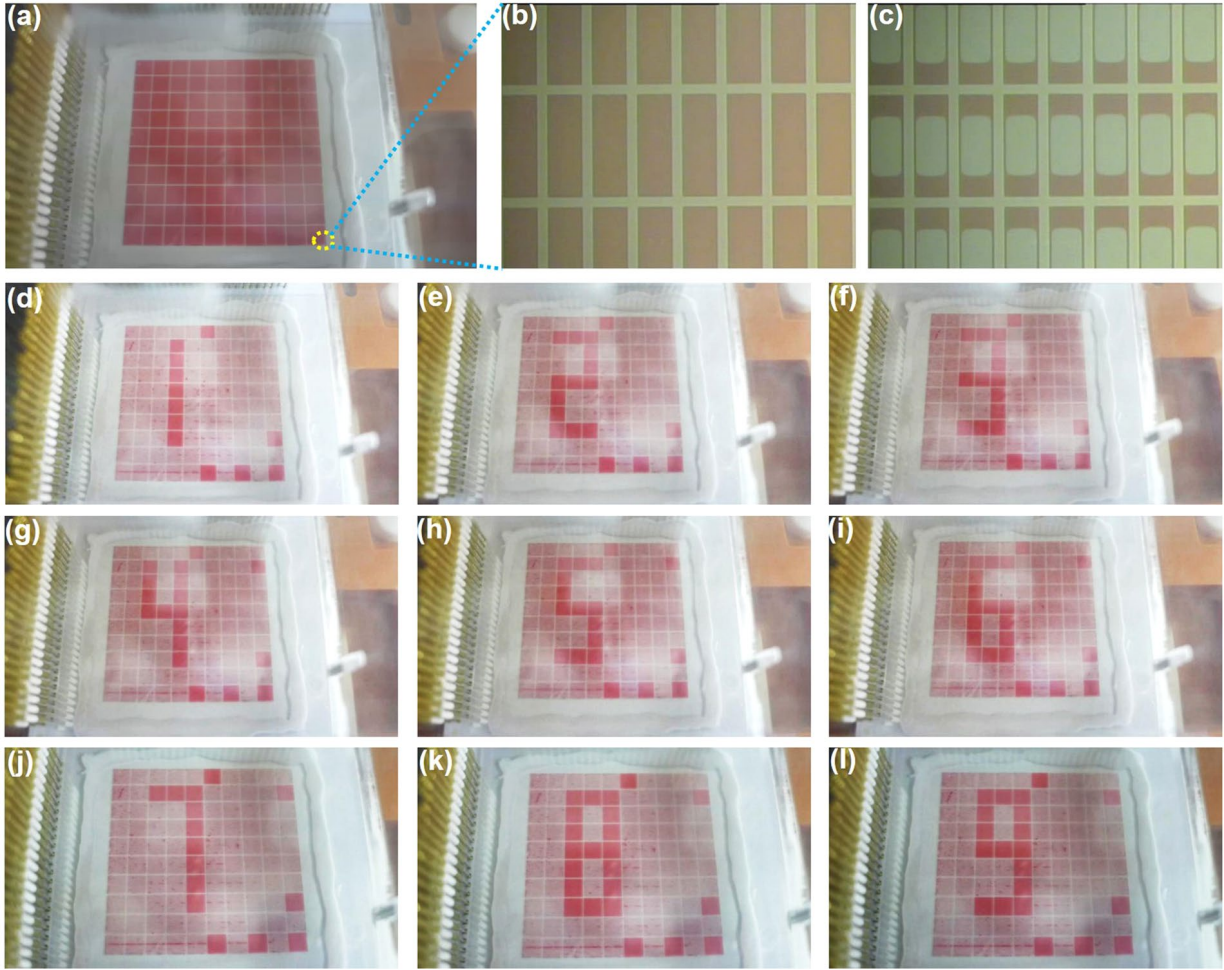
(**a**) Photo of the fabricated EWD sample with 10 × 10 pixel array. (**b**) Zoom-in image of one pixel when switched off and (**c**) on. (**d**∼**l**) Photos of the displayed numbers 1 to 9 respectively. Also see Supporting information S3.

Now we concluded that the SU-8 grid can be patterned on CYTOP with excellent adhesion and no compromise of its hydrophobicity by using our proposed process flow, which is very promising for EWD devices fabrication. To demonstrate the application of our proposed fabrication process, an electrowetting display sample with 10 × 10 pixel array was fabricated as shown in Fig. 6a. Each pixel contains an array of 100 μm × 300 μm sub-pixels in 30 rows and 12 columns (Fig. 6b and Supporting information S3). The lateral thickness of the SU-8 wall in one pixel is 30 μm. Since the purpose is to demonstrate the simple and robust fabrication process, these pixels were not fabricated on a thin film transistor (TFT) backplane. The 100 pixels were controlled individually by a homemade external circuit (Supporting information S3).The oil in the grid of one pixel contracted when a 13 V voltage was forced to the electrode of this pixel. Once the voltage was switched off, the oil fully recovered. The contraction and recovery of the oil was recorded by a CCD camera with video frequency of 25 frames/s (Supporting information S3 and Video-2). Both the contraction and recovery of the oil finished within one video frame which indicates the response time of the EWD is less than 40 ms. The EWD performance was mainly decided by the hydrophobicity of the surface, thus the measured EWD effect confirmed that the hydrophobicity of the CYTOP was well maintained in our fabrication process. By controlling the input signals of the pixels one by one, dynamic images of number 1 to 9 were displayed (Video-3). These results indicate the fabricated EWD is pretty good and possible for video frequency image display when combined with a TFT backplane. The fabrication of a EWD sample on a TFT backplane by our proposed simple and robust process is ongoing in our group.

In the above fabrication process, SiO_2_ was used as the sacrificial layer. However, the sacrificial layer was not limited to SiO_2_. Other kinds of oxides or metals (e.g. Ti or Au) can also be deposited onto CYTOP for sacrifice. This gives us additional freedom for the device fabrication. Since the process is simple and the hydrophobicity of the exposed surface of the fluoropolymer was maintained with excellent adhesion of the stack, we believe this strategy is easily repeated in different labs and can be generalized to large area substrates. Furthermore, the sacrificial layer strategy is compatible with the existing manufacturing process of EWD, which is good for cost control.

In summary, a simple, robust and manufacturing compatible photolithography process on highly hydrophobic surface was developed in this paper. By introducing a sacrificial layer between the photoresist and the fluoropolymer layers, the fabricated patterns was found adhere to the highly hydrophobic surface excellently and the hydrophobicity of the exposed highly hydrophobic surface was maintained. EWD devices were fabricated following our developed fabrication process, which shows dynamic image displaying with response time less than 40 ms. These results demonstrate our developed fabrication process is very promising for EWD manufacturing.

## Methods

Spin coating of CYTOP and photoresist was using a CEE^**®**^-200 (Brewer Science^**®**^) spin coater. SiO_2_ was deposited by e-beam evaporator (K. J. Lesker AXXIS). Contact angle was measured by Dataphysics OCA20. Film thickness was measured by HORIBA UVISEL FUV ellipsometer and AMBiOS XP-1 profilometer. The adhesion of the fabricated microstructure to the substrate was characterized by using a standard tape test knit and a homemade bending equipment (Supporting information S2). EWD samples were fabricated on ITO coated glass substrate. The bottom ITO electrodes were first patterned by photolithography and wet etching. Then the SU-8 grid was fabricated following our sacrificial layer strategy. The colored oil (dodecane) was dosed by the reported self-dosing process^13, 23^ and then sealed under water using epoxy. The sample was tested for image display on a homemade test fixture (shown in Fig. 1d and Supporting information S3).

All data generated or analysed during this study are included in this published article (and its Supplementary Information files).

## Electronic supplementary material

Supplementary info

Video-1

Video-2

Video-3
