# Supplementary material for: A sacrificial layer strategy for photolithography on highly hydrophobic surface and its application for electrowetting devices
Source: Sci Rep. 2017 Jun 21;7:3983. doi: 10.1038/s41598-017-04342-z (PMC5479844; doi:10.1038/s41598-017-04342-z)
Supplement: Supplementary file 1 — Supplementary info [file 41598_2017_4342_MOESM1_ESM.pdf]

Supporting Information

A sacrificial layer strategy for photolithography on highly hydrophobic surface and its application for electrowetting devices

Han Zhang[1], Qiuping Yan[1], Qingyu Xu[2,4,*], Changshi Xiao[3,4,*], Xuelei Liang[1,*]

[1]Key Laboratory for the Physics and Chemistry of Nanodevices andDepartment of Electronics, Peking University, Beijing 100871, China

[2]Department of Physics, Southeast University, Nanjing 211189, China

[3]School of Navigation, Wuhan University of Technology, Wuhan 430070, China

[4]Nanjing Jingaowei Optoelectronic Technology Co. Ltd., Nanjing 210007, China

S1 Detailed information for photolithography

As mentioned in the main text, the water contact angel (CA) of the hard baked CYTOP film is ~115°, which is too hydrophobic for spin coating of photoresist (Fig. S1.1 a). It was reported that SU-8 resist can be coated on soft baked CYTOP film because the surface energy of CYTOP was not lowered to its minimum value of ~20 mN/m only by soft bake.[1] However, this strategy is not robust. The solvent of CYTOP solution is very volatile and we found the surface energy of CYTOP drops very quickly as it drying even at ambient temperature. After several minutes of the CYTOP film coating, the SU-8 3010 resist (high viscous) cannot be uniformly spin coated. Usually the SU-8 3010 resist that dropped on the substrate will spin off the substrate when it began to spin. Occasionally pretty good spin coating of SU-8 was obtained on the un-baked CYTOP surface (Fig. S1.1 b). However, we found it depends on the batch of the SU-8 resist we bought. Even the SU-8 resist can be coated on CYTOP, it shrinks during the soft bake (95°C) before exposure, leaving a large blank area (Fig. S1.1 c) and results in very thick edge of the resist film (Fig. S1.1 d). Though microstructures can be obtained after exposure and develop of the resist in the left-over area, the adherence of the structure to the substrate was extremely poor which can be blow off by nitrogen gun (Fig. S1.2). Following our sacrificial layer

strategy, both high viscous resist (SU-8 3010) and low viscous resist (S1813) can be spin coated uniformly on rigid and flexible substrates (Fig. S1.1 e-h).

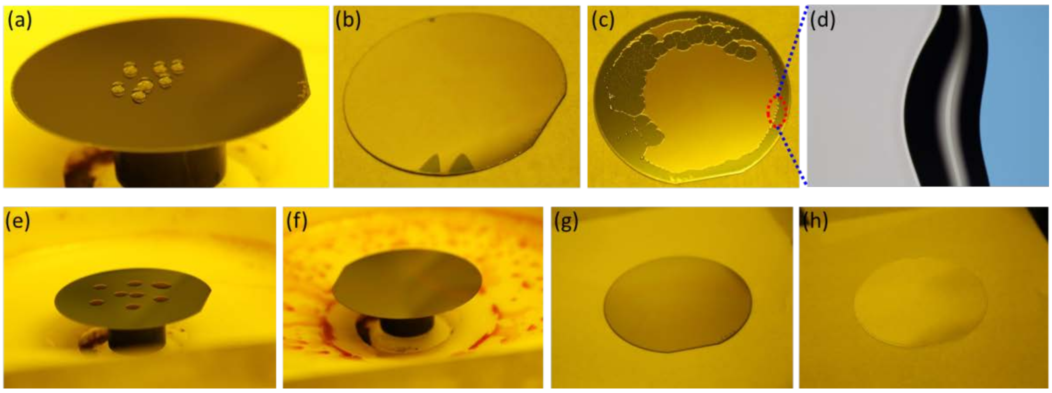

Fig. S1.1 (a) Water drops on hard baked CYTOP, which demostrate the surface is highly hydorphobic. (b) SU-8 can be coated on soft baked CYTOP occasionally, but it shrinks during soft bake of the resist (c). (d) Zoom-in image of the shrinked edge of the resist. (e) S1813 droped on SiO_2/CYTOP and spin coated uniformly (f). (g) SU-8 3010 coated on Si wafer and (h) PET substrate uniformly.

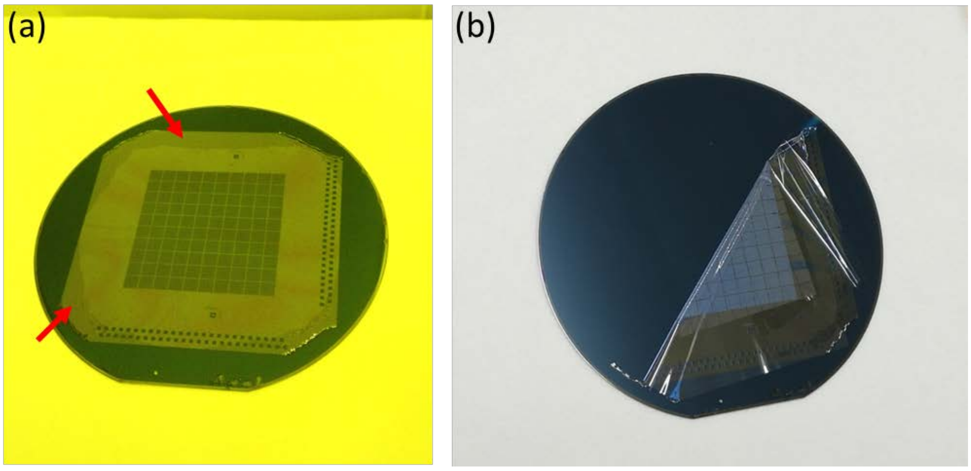

Fig. S1.2 Though SU-8 can be coated on CYTOP directly as shown in Fig. S1.1 (b), the fabricated microstructures do not adhere to the substrate well. (a) The fabricated SU-8 structures exfoliated from the edge during developing, as shown by the arrows. (b) The structure was blown off by strong nitrogen flow.

The SU-8 resist was spin coated with 100% coverage and very minor edge bead effect (Fig. S1.3 a-c) when sacrificial layer was employed. Photolithography is very easy due to the good resist coating and the results on both rigid and flexible substrates were shown in Fig. S1.3 d-i.

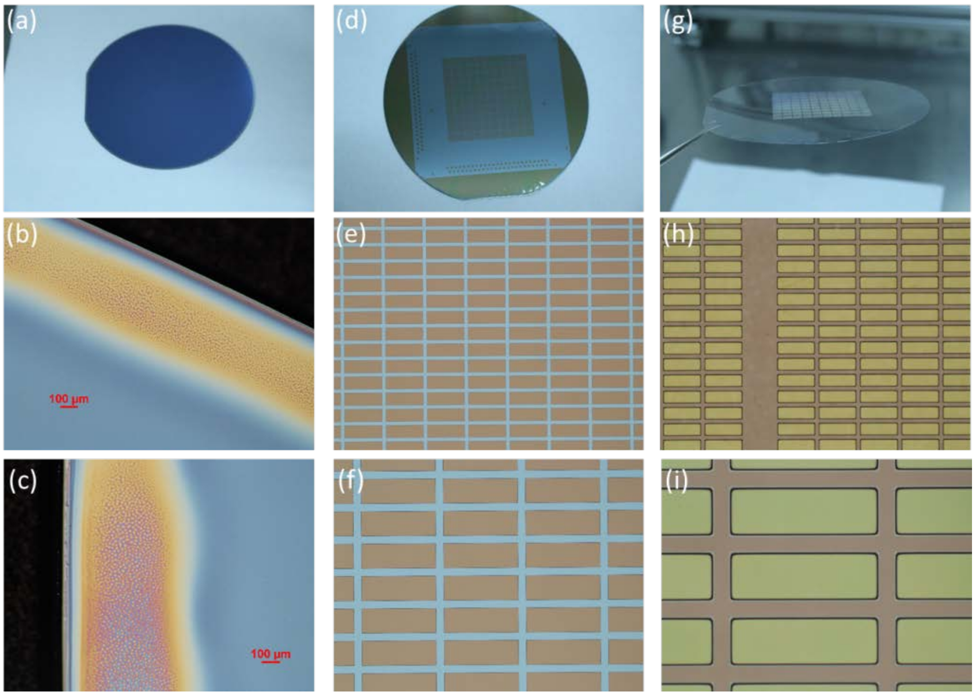

Fig. S1.3 (a) Uniform SU-8 coating. (b, c) Zoom-in images at the wafer edge which showing minor edge bead effect. (d) Exposed SU-8 pattern on Si wafer and (e,f) zoom-in images. (g-i) Corresponding results on PET substrate.

After exposure, the exposed SiO_2 was cleanly etched to expose the surface of CYTOP. Then the sample was annealed and the obtained structure adheres to the substrate well (Fig. S1.4).

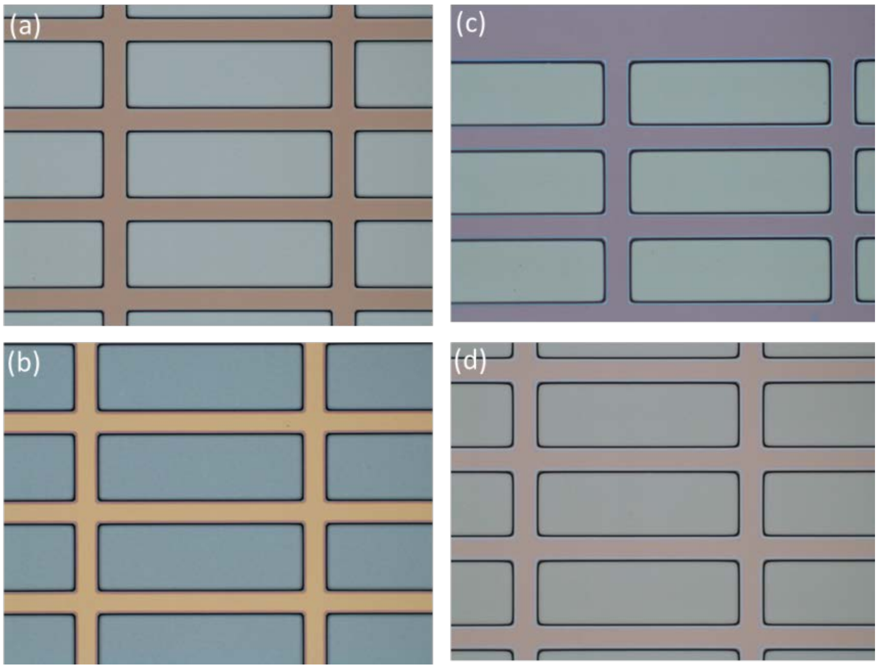

Fig. S1.4 Typical images of finally fabricated structures on (a, b) Si wafer and (c,d) PET substrates.

S2 Adhesion test

The standard tape test[2] was used to characterize the adhesion of the evaporated SiO_2 to CYTOP. The as deposited SiO_2 and hard baked CYTOP were cut into about 1mm × 1mm squares. Fig. S2.1a shows the photos of test results of the SiO_2 on CYTOP. Fig. S2.1b shows the adhesion of 150 ^{o}C annealing treated SiO_2 to CYTOP. These results confirmed the SiO_2 adhered to CYTOP very well.

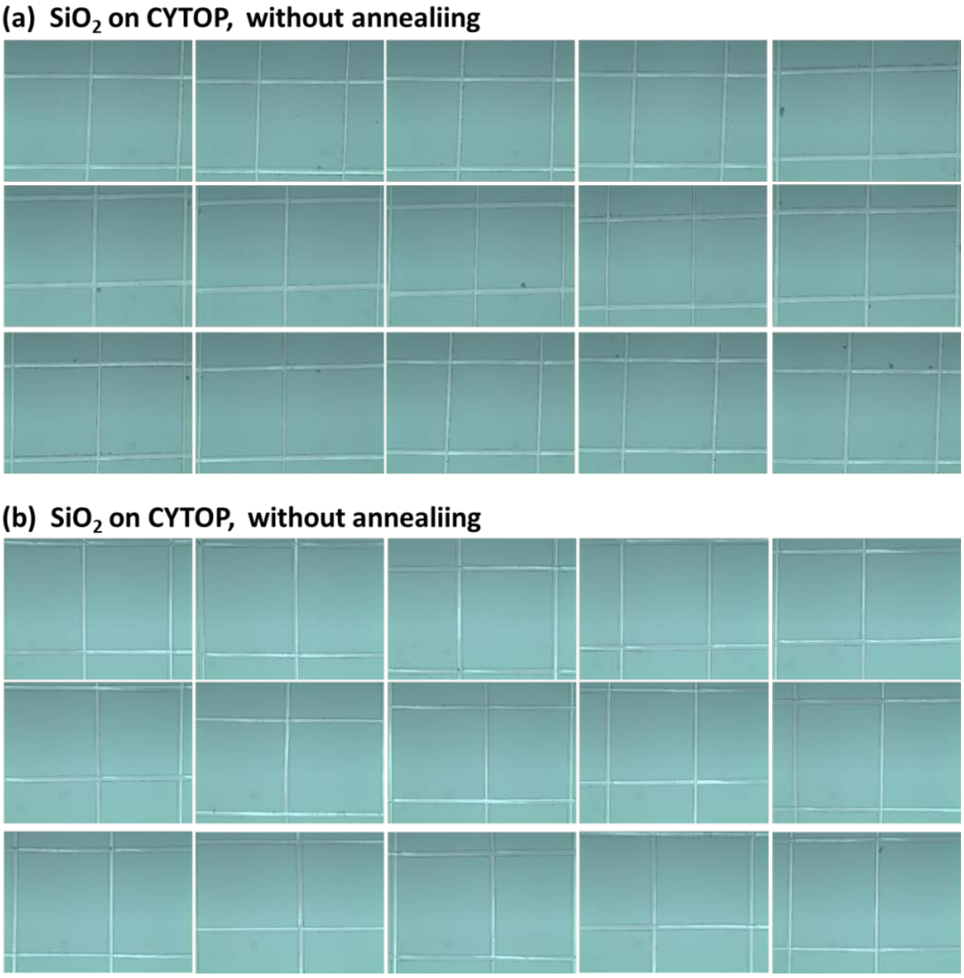

Fig. S2.1 Photos of ahesion test of SiO_2 to CYTOP that were taken at different locations aroud the wafer

The adhesion of the finally fabricated grid structures to the Si wafer was also tested

by tape test (Fig. S2.2).

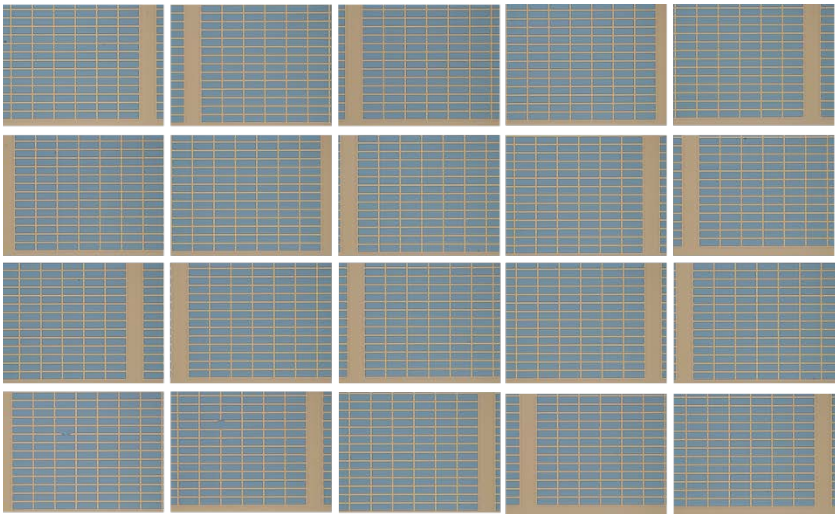

Fig. S2.2 Photos of the grid structures on Si wafer taken at different locations after tape test.

The file of video-1 shows the bending test for characterizing the adhesion of the microstructure to CYTOP on flexible substrate. The structure was not peeled off even in the most strained area after 10,000 times bending (Fig. S2.3), which indicate an excellent adherence was obtained following our strategy.

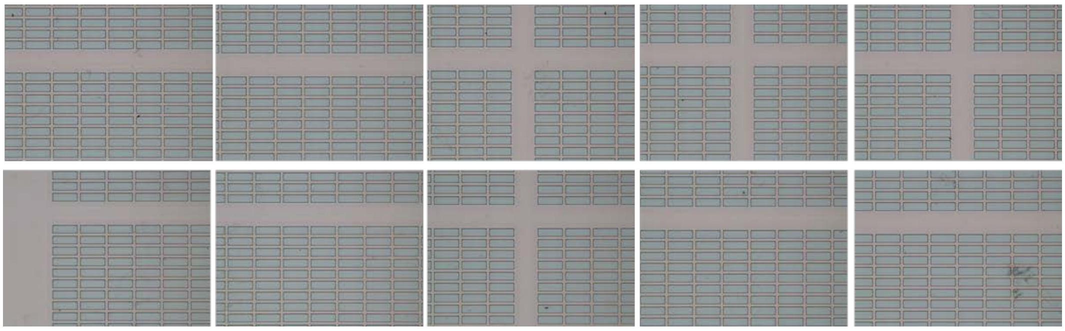

Fig. S2.3 Photos of SU-8 grid on PET after bending test.

S3 Fabrication and test of electrowetting display samples

To fabricate EWD samples, 0.5mm thick glass with 80 nm ITO were used for the bottom and top substrates. The bottom electrodes were patterned by photolithography and wet etching of ITO. Then CYTOP were coated and SU-8 grid was fabricated following the sacrificial layer strategy. Then dodecane with red dye were dosed by the self-dosing method.[1] The top substrate was sealed under water by using epoxy. The

samples were tested by using a homemade test fixture and external circuit (Fig. S3.1). The signals of image was firstly feed to the control module from computer and then to the EWD smple. The relatively slow signal feeding makes the image changes slow. However, the switching of sub-pixles in each pixel of the 10×10 pixel array is very fast.

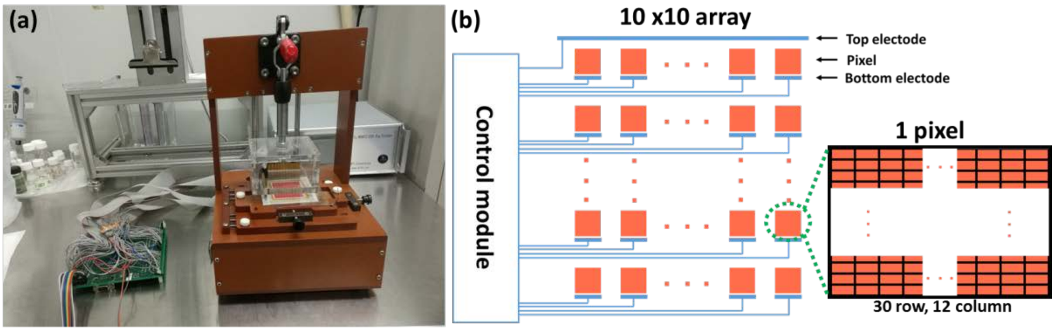

Fig. S3.1 (a) home made test fixture and external circuits. (b) Control diagram of the 10×10 pixel EWD sample. The pixels are controlled individually and rough dynamic image can be displayed.

The file of video-2 shows the switching on and off of an individual pixel with the control signal shown in Fig. S3.2

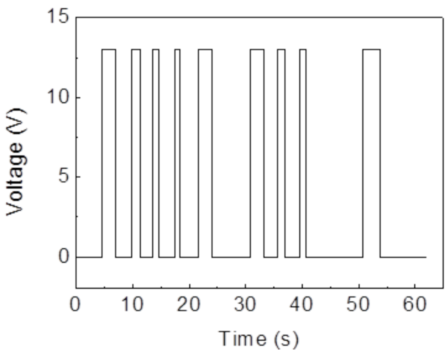

Fig. S3.2 Control signal of one pixel corresponding to video-2 and Fig. 6 b,c in the main text.

The file of video-3 is shows the dynamic displaying of number 1 to 9.

Reference:

1 Zhou, K., Heikenfeld, J., Dean, K. A., Howard, E. M. & Johnson, M. R. A full description of a simple and scalable fabrication process for electrowetting displays. *J Micromech Microeng* **19**, doi:Doi 10.1088/0960-1317/19/6/065029 (2009).

2 *Standard Test Methods for Measuring Adhesion by Tape Test, ASTM D3359 - 09, https://www.astm.org*.
